# Duration‐dependent Effects of Diet on Cognition and Gut Microbial Composition in an Aging Rat Model

**DOI:** 10.1002/alz70860_104514

**Published:** 2025-12-23

**Authors:** Rebecca J Solch‐Ottaiano, Madison Prats, Malaika Subramanian, Nicholas Prus, Irene Yu, Elizabeth Engler‐Chiurazzi, Gregory J Bix, Demetrius M Maraganore

**Affiliations:** ^1^ Tulane University, New Orleans, LA, USA

## Abstract

**Background:**

Dietary adherence is associated with the risk of cognitive decline and Alzheimer's disease. A Western diet (WD) is associated with increased risk, while a Mediterranean diet (MeDi) is associated with decreased risk. The gut microbiota may play a role in mediating this risk as it is modulated by diet. However, results from animal models used to study mechanisms of diet‐induced cognitive dysfunction are variable which may be in part due to the different study duration. The objective of this study was to determine the longitudinal effects of diet on cognition and gut microbiota in an aging rat model.

**Method:**

Ten‐week‐old Male Fischer344 (F344) rats were randomized to a MeDi or WD (*n* = 8/group). Morris water maze (MWM) was completed at 3.5, 9.5, and 15.5 months of diet to test learning and memory. Fecal samples were collected prior to behavioral assessment and analyzed via 16s rRNA sequencing.

**Result:**

Body weight did not differ between the MeDi and WD groups. At 3.5 and 9.5 months there was no difference in latency in the MWM. However, at 15.5 months the MeDi rats had a shorter latency relative to WD rats. Regarding microbial composition, by 3.5 months there were distinct diet‐dependent compositions as indicated by beta‐diversity measures. At the taxonomic level, there were 32, 19, and 25 differentially abundant taxa at 3.5, 9.5, and 15.5 months, respectively. For all time points, the phyla *Actinobacteria* and *Desulfobacterota* were lower in MeDi rats while *Firmicutes* was higher in MeDi rats relative to WD rats. Additionally, the Firmicutes to Bacteroidetes ratio, associated with gut homeostasis, was lower in the MeDi rats relative to the WD rats at 3.5 and 15.5 months of diet. Genera that differed at only 15 months included *Bilopha, Candidatus Saccharimonas, Defluviitaleaceae UCG‐01, and Anaerotruncus*.

**Conclusion:**

Cognition was not impacted by diet until 15.5 months, indicating that the duration of a diet should be considered. Additionally, there were subtle differences in microbial taxa between time points as animals aged and duration increased. The differentially abundant bacteria at 15 months, coinciding with the time point of reduced cognitive performance, may serve as potential microbial biomarkers related to cognition.

